# The future of Diabetic Kidney Disease management: reducing the unmet need

**DOI:** 10.1007/s40620-020-00820-2

**Published:** 2020-08-04

**Authors:** Dick de Zeeuw

**Affiliations:** Department of Clinical Pharmacy and Pharmacology, University of Groningen, University Medical Centre Groningen, De Brug 40-1-026, EB70, Postbox 30001, 9700 RB Groningen, The Netherlands

**Keywords:** Diabetes, Clinical trials, Kidney, Response variation

## Abstract

Patients with type 2 diabetes run a high risk for progressive renal function loss. Many interventions have been tested to reduce the risk, but we are nowadays still confronted with a high unmet need. To improve on this unmet need, we will have to change the current strategies in drug discovery, clinical trials and clinical practice. Target finding and the search for new interventions has to change to include more individual mechanistic approaches. Drugs will be selected on basis of finding the “individual” mechanism of renal function loss by looking at renal tissue biopsies or new biomarkers in urine or plasma. To test the promising drugs for clinical efficacy and safety and reduce the unmet need, trial design in type 2 diabetes will have to alter. First, selection of patients at risk for progression of renal function loss will need to be more specific. True progressors need to be identified by switching from classical risk determinants (low eGFR and high albuminuria) to new surrogates like steep eGFR slopes. In addition, the investigational drugs should only continue into registration trials in responder populations: patients that show a good response in the target/surrogate risk marker and no bad responses. This way we will improve the success of hard outcome trials, which has been poor in the past decade. We will reduce the unmet need and reduce the number of patients that are exposed to long term trial treatments without any benefit or even harm. Platform design and basket trials will catch the non-responders and switch them to other investigational drugs with different mechanism of action.

Drug registration will be much more directed to the individual patients and will lead to improved individual patient medication advices and improved individual efficacy and safety. We are entering the era of precision medicine in nephrology.

## Introduction

**Fig. 1:**
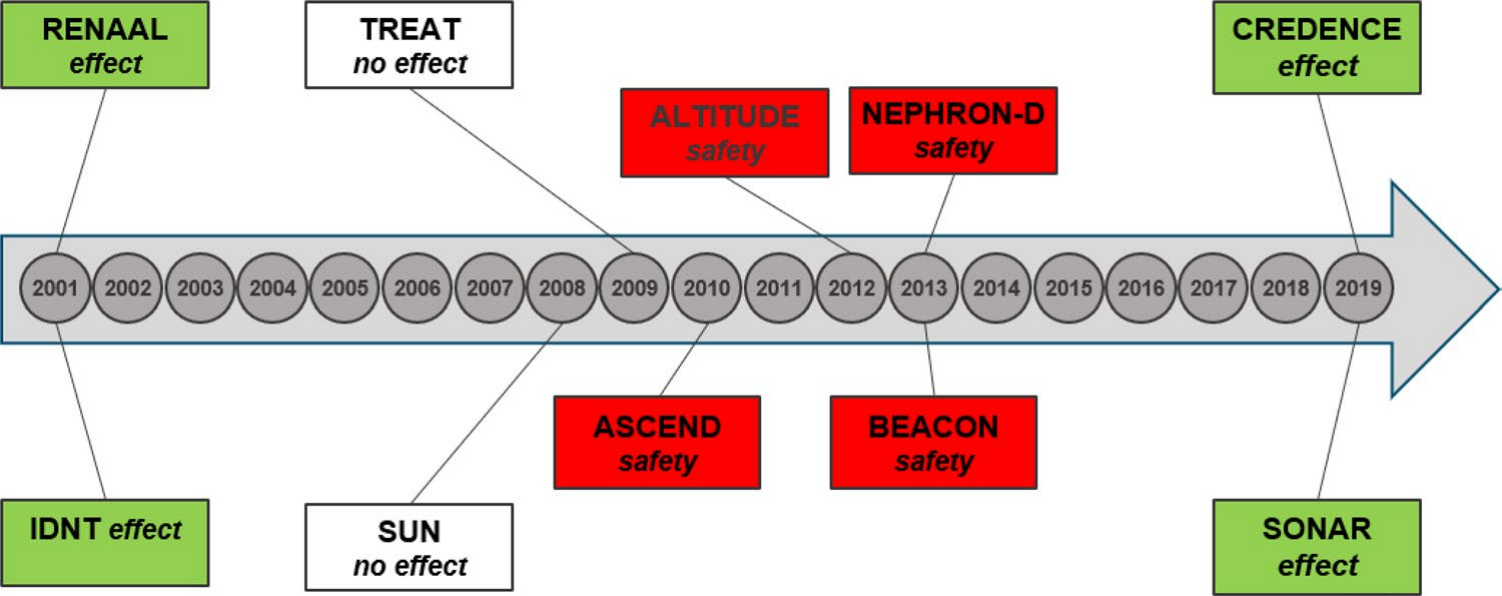
Clinical trials testing new drugs for renal/CV protection in type 2 diabetes with nephropathy: nearly 20 years of no success due to stopping of trials for safety reasons or due to no success on the surrogate or hard endpoint [1–10]

Diabetes is a growing disease with high renal morbidity and mortality. Despite multiple efforts over the last decades to find therapies to halt the progression of renal disease in type 2 diabetes, success remains limited and renal protective guideline therapy remains largely based on inhibition of the renin-angiotensin-system (RAS) [1–8]. Recently the renal community was thrilled by the successes of SGLT-2 inhibitors and endothelin antagonist (Fig. 1) [9, 10]. However, without taking away this success, the unmet need in the population at risk remains extremely high. Are we doing something wrong in our approach to reduce the renal risk in type 2 diabetes? This review addresses the question whether we are using the wrong strategy for our target and drug finding, and/or whether we are using the wrong trial design in which we test new drug discoveries, and tries to formulate possible solutions.

## Does the current strategy “induce” unmet need?

### Target finding

To date the search for new treatment strategies to stop the progression of renal function loss in type 2 diabetes is based on targeting multiple different risk factors. Clearly the obvious target in diabetes is, and has been, high glucose. However, many other factors appear to play an important role as renal risk factors, e.g., lifestyle, glucose, blood pressure, cholesterol, body weight, smoking, albuminuria, potassium. High levels of these factors can be mechanistic in contributing to the increased renal risk in type 2 diabetes. Another way of looking at mechanisms for progressive renal function loss is analysing renal biopsy tissue of the type 2 diabetic patient. It was long believed that progressive loss of renal function in diabetic patients was the effect of diabetes itself on the kidney since renal lesions were found to be typical. However, in type 2 diabetes renal lesions may present also as non-diabetic lesions matching other glomerular renal disease [11].

Thus, the search for a specific target to halt the progression of renal function loss in type 2 diabetes appears to be complicated due to the heterogenicity of the disease both in phenotype and in renal tissue pathology. This alone might explain why we have such high unmet need in the trials with new investigational drugs that target the whole type 2 diabetes population and mostly target a single risk factor.

### Target response

To intervene and lower the high risk of progressive renal function loss in patients with type 2 diabetes, we have successfully searched for and developed drugs that work on lowering the risk factors glucose, blood pressure and cholesterol. Recently we have also added albuminuria as a potential target for therapy [12].

To test the target response for renal protection the investigational drug is tested for effectively lowering the risk factor as a surrogate marker in one or more phase II trials. If yes, this is followed by a phase III trial in which the drug is tested for protecting the kidney based on hard renal outcomes like end-stage renal disease (ESRD) expressed by the need for dialysis or renal transplantation. Indeed, new drugs that successfully lowered glucose, or blood pressure, or cholesterol, or albuminuria (or a combination of them) have been further tested in phase III hard outcome trials. However, the success of advancing a surrogate marker to the phase III stage is based on a predefined mean effect size of the drug in lowering the surrogate marker in the studied population. This effect may not be true for each individual patient. In fact, there is high interindividual variability in the response of surrogates like glucose, blood pressure, cholesterol, or albuminuria. This variability is such that the drug intended to lower the surrogate, may induce no effect or even an increase [13]. Thus, phase II surrogate response is highly variable. With respect to phase III outcomes, reduction of renal risk, a similar high variability is observed. Although we cannot look in these phase III trials at an individual level of variability, either you reach an endpoint or not, the mean renal risk lowering is variable. Successful phase III outcome trial like RENAAL, IDNT, SONAR and CREDENCE never show 100% risk reduction compared to placebo, but we are already thrilled if we reach a decrease between 20 and 30% renal risk reduction, leaving more than 70% in renal risk!

Most intriguing of the above findings is that the variability in the surrogate marker response triggers a phase III study design in which all patients (responders and non-responders) are enrolled. Post hoc analysis of phase III trials show that the group of patients that benefits most from the investigational drug with a lowering of their renal risk is the group that shows the most effect on the surrogate marker. RENAAL, a positive phase III trial, showed a great correlation between short term investigational drug induced albuminuria change and the change in ESRD risk: indeed those with a fall in albuminuria appear to be renal protected, but more than 50% show no change or an increase in albuminuria which is associated with no change or an increase in renal risk [14]. The overall effect in the tested population was a significant lowering or renal risk, a positive trial outcome, leading to registration of the losartan. However, RENAAL did have a high unmet need! This variability in response can even lead to trial result that are negative for the whole tested population, whereas a subgroup does benefit from the drug. A post hoc analysis of a negative trial like ALTITUDE testing the addition of a renin-inhibitor to guideline therapy, showed significant renal protection in a group of patient in which the investigational drug lowered the target (albuminuria) more than 30% [15]. However, most patients had less than 30% albuminuria reduction and that group showed an increase in renal risk, totalling to a negative trial.

In summary, current target response in treating type 2 diabetes patients to slow their progressive renal function decline is, just like target finding, very heterogeneous. The response of the surrogate marker to the investigational drug is highly variable as is the effect of the drug in reducing renal risk measured in hard renal outcome parameters. The variability in the lowering of the surrogate marker is related to the variability in ultimate renal protection. No surprise that the current approach of measuring target response creates a setting that, even in a statistically significant trial, ends with approving a drug that only protects part of the type 2 diabetic population and leaves a high unmet need.

### Off-target response

The current approach in drug development and drug registration for renal protection in type 2 diabetic patients has the rule that the drug and the required dose is developed for lowering of the single surrogate marker, the target. However, most drugs have more effects than the target effect, e.g., angiotensin receptor blockers (ARB) affect not only blood pressure but at least 10 other parameters [13]. These so-called off-target effects are usually categorized in the safety paragraph of drug registration trials. However, these off-target effects may influence the effect of the drug on the renal outcome. A good example is the fact that ARB may increase serum potassium, which in turn has its effects by increasing the risk of progressive renal function loss [16, 17]. Intriguingly, those off-target effects show marked individual variability. Thus, although ARB’s do lower blood pressure and do lower albuminuria, both helping in protecting the kidney, they may offset that protection by their off-target effects on potassium. In ALTITUDE it is likely that dual blockade of the RAS by the addition of the renin-inhibitor to guideline therapy of angiotensin converting enzyme inhibitors (ACEi) or ARB’s did induce a slightly more reduction in blood pressure and albuminuria, but had a big effect on further increasing serum potassium, with an end result of a negative trial [18].

In summary, the on-target and off-target responses show both high interindividual variability, and this can impact on the renal protective effect of an investigational drug resulting in mitigation of the potential renal protection resulting in unmet need.

### Trial design

Phase III trial design carries a bias that contributes to potential unmet need in clinical practice. In fact, the design of a current clinical outcome (registration) trial is a mismatch when compared to our clinical practice in which we select and dose a drug to individual needs of a patient. We change the drug or change the dose in a patient if we see no or too little response of the surrogate marker, or too many off-target effects. In type 2 diabetes trial design we have usually fixed doses and keep every patient in the trial irrespective of whether the drug lowered the surrogate or has an unwanted off-target effect. This way, we have a big chance of doing phase III trials that do not reach the preselected criterium of a certain amount of renal risk lowering, resulting in negative trials, and no registration of new investigational drugs. The selection of drugs that will be registered for clinical practical use is limited to those drugs that show a (big) effect in the majority of patients, but it will deny registration of drugs that may be very renal protective in a sub selection of the type 2 diabetic patients.

### Future strategies to reduce unmet need

Now that we understand partly how the current target, drug finding, and trial design have a big impact on the current high unmet need in protecting the kidney in type 2 diabetes patients, we need to find out whether we can do something about this in the future.

### Target and drug finding

Drug intervention should not be only aimed at a single risk marker from a list of potential risk markers but should look much more at the underlying mechanism(s) of progressive renal function loss. We now know that the progressive renal function loss in type 2 diabetes can be caused by vary different mechanisms. The success of multifactorial intervention approaches [19, 20] is indirect evidence that we will need to solve the unmet need by identifying the which mechanism plays a role in which patient. To find the right therapy for the right patient, we will need to look for mechanism(s) in each single patient and establish whether there are commonalities so that we can design effective drugs for a selected group of patients. Renal biopsies are highly wanted for such an approach. Only then can we start looking for drugs that will intervene in the renal progression mechanism that is playing in that patient. This search should be carried out at each stage of the disease, early as well as late since it is likely that the mechanism of progressive renal function loss changes over the stages. Thus, successful preventive therapies may differ considerably from successful intervention therapies. Several different world-wide consortia are currently collaborating to tackle this problem of this type of target finding. The BEAt-DKD consortium is a good example. It strives to define a new single or set of biomarker(s) that predicts the risk of renal function loss in a patient with type 2 diabetes. Renal biopsies, renal imaging, proteomic, metabolomics, genotyping, and full system biology are developed to understand the (individual) mechanism of progressive renal function loss and the important targets for intervention [21].

When we have a potential mechanism for renal disease progression in a group of type 2 diabetic patients, drug finding for this group is the next step. This appears to work successful with the current strategy, with good examples of interventions in the angiotensin system (ACEi and ARB), and recently in glucose metabolism [Sodium Glucose Transporter-2 (SGLT-2) inhibition]. Even though we have developed drugs targeting a putative mechanism of disease the success of those drugs appeared to be not always based on that mechanism only. ACEi and ARB do not only protect the kidney by lowering the activation of the RAS, as does SGLT-2 inhibition not only protect the kidney due to changes in glucose metabolism [22]. Drug finding can be further optimized by better understanding the complexity of the interaction between different mechanisms of disease and the complexity of the actions of a single drug on these mechanisms.

### Trial design

Given the high residual risk for progressive renal function loss in the current please III trial results, the question remains whether the current trial design is effective to reduce that residual risk. Should we not re-orientate ourselves and start thinking that we will deliver much more renal protection for our patients by approaching trial design on a more individual patient basis? Patient selection could help a lot.

### Only patients that show progression

Phase III trials are time consuming and very costly. This has to do with the fact that renal endpoints like reaching ESRD take time, and one needs many trial endpoints to obtain enough power for proving the effect of the drug on renal risk. Therefore, we select patients in such trials that have a high risk for reaching such an endpoint. Enriching trial enrolment for high renal risk is done currently by two parameters: low eGFR and high albuminuria. The level of those two parameters appears to be the most important in defining a patient’s renal risk. Patients with low eGFR and patients with high albuminuria run a very high risk [23]. However, the relation between low eGFR or high albuminuria and the high risk for ESRD can only be established in analysis of groups of patients: a group of patients with a high level of albuminuria have more chance of an ESRD event. Could it be that there are still individual patients with high albuminuria that have little risk or no progressive renal function loss and thus low risk for a renal endpoint? If true, then phase III trial are populated with low risk patients that will not progress to ESRD and thus cannot be used to test whether a drug lowers the risk. In other words, there will be a lot of seemingly unmet need (one cannot test the effect of an antibiotic if the patient is not infected)!

Recently other renal endpoints have been suggested for use in phase III trial among them the slope of the eGFR. This parameter is good reflector of the progressive renal function loss of an individual patient. To know whether high albuminuria or low eGFR are the right selection criterium for renal risk, we should find out whether each patient with high albuminuria and low eGFR has a steep slope of eGFR loss. There are many publications on the strong relation between the level of albuminuria/proteinuria and eGFR slope. But none of them show individual data. In those cases where individual data are plotted one can observe that many diabetic and non-diabetic patients with high proteinuria or low eGFR can have low eGFR slopes, in other words they are non-progressors [24]. A post-hoc study of the RENAAL trial, in which the authors were able to collect eGFR data of a subset patients before they entered the trial itself [25] confirmed this finding (unpublished).

In conclusion, to avoid trial failures and results with high unmet need, we need to find better tools to select the patient that has the “disease” and runs true risk of progressive renal function loss. This would not only tackle the unmet need discussion, but it would also increase the power of the trial since the risk for the endpoint ESRD would increase markedly. This would increase the effect size and thus the number of needed patients, and will reduce the duration of the trial. Selecting patients on their eGFR slope (e.g., > 3 ml/min/year/1.73 m^2^) would be a much better approach than the current renal risk estimation by eGFR and albuminuria level.

### Only patients with surrogate response

Selecting patients on their short-term response to the investigational drug can further help to reduce the unmet need in the long-term phase III clinical trials. This is supported by a recent post-hoc metanalysis of many previous renal outcome studies showing that those patients that show a short-term (first couple of months) lowering of the surrogate marker, e.g., albuminuria, have much more long term renal protection than those who did not [12]. The SONAR trial looking at the effect of the endothelin antagonist atrasentan on renal outcome has looked at this approach by a design in which the patients were preselected on the bases of an enrichment period of 6 weeks in which the effect of atrasentan was measured in each individual both with respect to on-target efficacy (albuminuria lowering) and off-target effects (sodium retention). Only patients that had more than 30% albuminuria reduction and no signs of sodium retention were enrolled in the long-term outcome trial [26]. The outcome of this SONAR trial is recently published and shows that despite glitches in the execution of the enrichment design, the approach could well help in reducing the unmet need. The responders showed one of the largest effect sizes of a phase III renal diabetes trial, 35% renal protection. This even though the trial was underpowered by a much too short observation time with very low event rates (all due to unexpected early trial stop [9]).

**Fig. 2:**
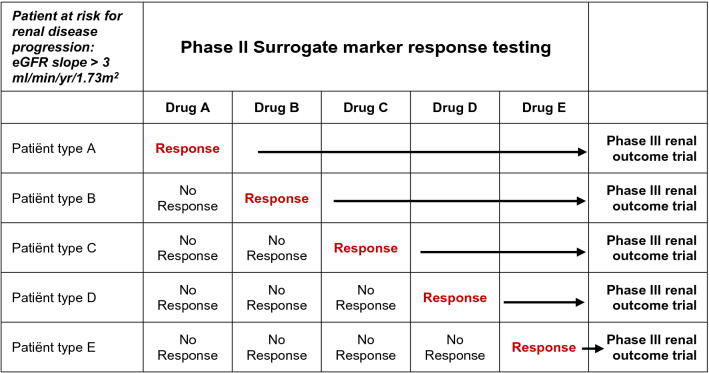
Future trial design: patient selection on eGFR slope > 3 ml/min/year/1.73 m^2^. Subsequent exposure of the patient to a combination of an platform and umbrella design in which each patient is tested for short term drug response in the surrogate marker. If successful, the patient continues in the phase III outcome trial; if failed the patient moves to the next level of another drug testing on short term effect

However, by selecting patients on their response we do reduce the unmet need in the trial, but we do remain with the non-responders for which the investigational drug does not work. To solve this problem, we can refer to our colleagues in other specialties like oncology who design trial platforms and basket trials in which the non-responders rotate to the next investigational drug with a different mechanism of action. These novel trial approaches are a new way in which we can test the renal protective effect of an array of different investigational drugs each of them having a different mechanism of action (Fig. 2). Ultimately this could result that we serve each patient by finding the right drug for the right patient. This type of so-called precision medicine is the ultimate solution in trial design for the high unmet need in protecting the kidney in patients with type 2 diabetes [27].

### Clinical practice

Finally, all these new approaches in drug discovery and trial design are interesting but can only be of value in reducing the unmet need if the drug then works in clinical practice for the individual type 2 diabetic patient. The ultimate proof will be real world trial designs that test the effect of these drugs outside the rigorous setting of the current phase III clinical trial design, but inside the normal daily routine clinical setting. Then the true unmet need might be met.
